# Myeloid cells, tissue homeostasis, and anatomical barriers as innate immune effectors in arterial hypertension

**DOI:** 10.1007/s00109-020-02019-1

**Published:** 2021-01-14

**Authors:** Johannes Wild, Philip Wenzel

**Affiliations:** 1 Center for Thrombosis and Hemostasis, University Medical Center Mainz, Mainz, Germany; 2 Center for Cardiology - Cardiology I and CTH Professorship “Vascular Inflammation”, University Medical Center Mainz, Langenbeckstr. 1, 55131 Mainz, Germany; 3 German Center for Cardiovascular Research (DZHK) – Partner site RheinMain, Berlin, Germany

**Keywords:** Innate immune system, Arterial hypertension, Essential hypertension, Immune system

## Abstract

Although essential hypertension affects a large proportion of the human population and is one of the key drivers of cardiovascular mortality worldwide, we still do not have a complete understanding of its pathophysiology. More than 50 years ago, the immune system has been identified as an important part of the pathogenesis of arterial hypertension. An exceeding variety of recent publications deals with the interplay between the numerous different components of the immune system and mechanisms of arterial hypertension and has substantially contributed to our understanding of the role of immunity and inflammation in the pathogenesis of the disease. In this review, we focus on myeloid cells and anatomical barriers as particular aspects of innate immunity in arterial hypertension. Since it represents a first line of defense protecting against pathogens and maintaining tissue homeostasis, innate immunity provides many mechanistic hinge points in the area of hypertension.

## Introduction

Arterial hypertension is one of the key drivers of cardiovascular mortality worldwide. Already in 2005, the global burden of disease study predicted a number of 1.56 billion adults with prevalent hypertension in 2025 [1]. Back in 1949, Page introduced the so-called mosaic theory of the pathophysiology of essential hypertension, claiming that not a single factor but a complex interplay of different organs and circumstances is part of the disease [2]. In addition to the endocrine system, kidney, heart, and vasculature, the immune system has been identified to be a major contributor to hypertension. First experimental hints were collected in 1960, when Grollman et al. identified antihypertensive effects of pharmacological immunosuppression in a murine model with partial renal infarction [3]. Since these days, hypertension research increasingly focused on the immune system resulting in the tempting theory of essential hypertension as an autoimmune disease [4, 5].

The first step of classifying the numerous different participants of the immune system is the subdivision into innate and adaptive immunity. Innate immunity represents the first line of defense against pathogens, responsible for maintaining tissue homeostasis and preventing microbe invasion [5]. In this review, we attempt to focus on the role of innate immunity in the mechanisms of essential hypertension discussing briefly the cellular components of innate immunity but also go beyond immune cells, looking at tissue homeostasis, inflammatory microenvironment, and anatomical barriers such as skin and gut. We therefore want to provide space for novel, emerging ideas and unexpected concepts that seem to be promising for further advances in hypertension research.

## Murine models of arterial hypertension

As the pathogenesis of essential hypertension remains poorly understood, all of the animal models, which are commonly used in hypertension research, are limited in their translational value for the human disorder. Whereas there are several excellent animal models for primary hypertension, the lack of understanding of the exact pathogenesis of essential hypertension inevitably only allows attempts to mimic aspects of the human disease as good as possible. We want to introduce briefly the two most common inducible models as they form the basis for the largest part of the studies, which are cited in this review. Detailed overviews over all the existing genetic and inducible animal models including their advantages, disadvantages, and translation to human hypertension can be found elsewhere [6–8].

Studies using an infusion of the blood pressure hormone angiotensin II (AngII) represent by far the largest share of all murine models. Nearly half of all National Institute of Health–sponsored research projects in the field of arterial hypertension use this model [9]. Patients with essential hypertension show lower blood pressure and improved outcomes when treated with RAAS blockers or inhibitors. However, this clinical observation does not prove the causation that RAAS activation is the most important underlying cause of primary hypertension. Nevertheless, blood pressure elevations seen in animals treated with a continuous infusion of AngII can resemble blood pressure values seen in uncontrolled human hypertension and even induce hypertension-related organ damage [10, 11]. Since it is inducible, the model offers the advantage that different species and genotypes can be used easily for studies and the dose as well as duration of the AngII treatment can be adjusted dependent of the experimental question. As human essential hypertension is clearly not solely depending on AngII, the massive pharmacological challenge of this model only mimics blood pressure effects of a dysregulated RAAS and therefore is not suitable to explain the complete, highly complex pathogenesis of essential hypertension.

In the DOCA-salt model, the combination of mineralocorticoid treatment with deoxycorticosterone (DOCA) and intake of high-salt (e.g., sodium chloride), sometimes extended by uni-nephrectomy, provokes hypertension resembling some features of human low-renin hypertension. Although elevated levels of DOCA can contribute to some rare human forms of hypertension, the most common human form of mineralocorticoid-dependent hypertension involves hyperaldosteronism, which is then one of the subsets of primary and not essential hypertension. The model was often used to mimic salt-dependent hypertension which was explained by salt-driven fluid retention [12]. This concept is more and more questioned [13, 14]; however, despite all legitimate criticism, the model still is valuable, especially for studies with a focus on mineralocorticoid receptor blockage and hyperaldosteronism.

The development and choice of reproducible and translational animal models is still one of the biggest challenges in biomedical research in general and in hypertension research in particular. So far, there is no individual model that can exactly recapitulate all key features of human essential hypertension and reproduce the pathogenesis of this complex disorder. For the interpretation of experiments and publications aiming to contribute to the understanding of essential hypertension, the limits of the studied animal model has always to be taken into consideration to estimate the translational value.

## Cell-mediated innate immunity

Since the 1960s, most immune cells in innate and adaptive immunity were described to have contributing roles in essential hypertension [15, 16]. Therefore, a detailed description of the published evidence for every single innate immune cell type would exceed the scope of this review. Nevertheless, we try to provide an overview of the most important recent findings and focus on key pathways of innate immune cells in essential hypertension.

In 2005, de Ciuceis et al. published about the link between innate immune cells and arterial hypertension in mice: They infused osteopetrotic mice (Op/Op)—a mouse strain deficient in macrophage colony-stimulating factor (m-CSF)—with AngII and found reduced systolic blood pressure values as well as a protection from vascular dysfunction in the absence of m-CSF [17]. Their findings indicated that m-CSF-depending cells play a substantial role in AngII-induced hypertension and vascular dysfunction. As m-CSF induces monocyte and macrophage colony formation from bone marrow precursors, these subsets were suspected to participate in the pathogenesis of AngII-induced murine hypertension. Later, we have shown that selective ablation of lysozyme M–positive myeloid cells attenuated AngII-induced endothelial dysfunction, vascular inflammation, and blood pressure increase. This phenotype could be restored by adoptive transfer of monocytes, but not of neutrophils or of monocytes deficient in the AngII receptor type 1 (AT1R) or gp91phox [18].

As tissue-resident macrophages are present in most (if not all) organ systems in the human body [19, 20], they have been under special investigation [21], and the M1-M2 concept of macrophage polarization [22] has been considered to play a role in arterial hypertension. AngII as one of the most prominent hormones in arterial hypertension has been shown to be a potent driver of the M1/M2 ratio towards the pro-inflammatory M1 phenotype [23, 24], In a small cohort of 45 hypertensive and 15 normotensive patients, Ji et al. described more Th1 and Th17 cells in hypertensive patients, which was accompanied by higher IFN-gamma and IL-17 levels [25]. As M1 macrophages are described to promote the induction of Th1 and Th17 cells, macrophage polarization could be a factor in the development of arterial hypertension. This tempting‚ “good cop/bad cop” concept for M1 and M2 macrophages in hypertension was challenged by Moore et al. when they described the accumulation of M2 macrophages in the aortic wall of AngII-infused mice [26] after 14 days of treatment with strong evidence for a shift of the phenotype during the treatment.

Besides monocytes/macrophages, neutrophils and dendritic cells (DCs) are main cellular parts of the innate immune system. Neutrophil counts are elevated in hypertensive patients [27, 28] and in murine models of arterial hypertension [29, 30], but it is not clear yet, if they really drive hypertension themselves or if they are driven by the sterile, low-grade inflammation smoldering in hypertensive individuals. In any case, unequivocal evidence of a decisive role of neutrophils in initiating or promoting hypertension is lacking. Dendritic cells (DCs) exert divergent and important roles, which are required for the orchestrated immune response. They are the most potent of all antigen-presenting cells (APCs), presenting phagocytosed antigens to cells of the adaptive immune system. Antigen presentation and secretion of co-stimulatory cytokines by DCs are crucial for the exact guidance of T cell activation, polarization, and recruitment. This bridging position between innate and adaptive immune system makes DCs an interesting hub in the immunology of hypertension [31].

Kirabo et al. published that proteins modified by oxidation by highly reactive γ-ketoaldehydes (isoketals) found in different murine models of hypertension accumulate in DCs. In their study, this accumulation drives the DC-mediated production of pro-inflammatory cytokines and T cell proliferation resulting in arterial hypertension [32]. DCs also seem to be crucial for the development of a hypertensive response in AngII-infused mice. Lu et al. investigated FLT3L^−/−^ mice lacking classical DCs. After application of AngII, the mice showed reduced blood pressure elevation and lower amounts of inflammatory cells in the kidneys [33].

Taken together, there are several sophisticated studies which focused on monocytes, macrophages, and dendritic cells in experimental hypertension, and many of them could show blood-lowering effects of interfering with one of these leucocyte subsets. Unfortunately, the translational value of these studies for human essential hypertension could neither be proven nor drive a novel therapeutic approach.

## Toll-like receptors

As the orchestrated immune response is crucial for host defense, but also for wound healing and maintenance of tissue homeostasis, the simple depletion of myeloid cells is not feasible for humans. Therefore, investigating (dys-)regulations in more specific inter- or intracellular pathways of innate immunity has been the topic of further studies. Fast recognition of invading microorganisms is of utmost priority for the organism. Widely shared molecular structures of pathogens, so-called pathogen-associated molecular patterns (PAMPs), and their direct activation of pattern-recognition receptors (PRRs) of innate immune cells are key components of the early innate immune response [34]. In contrast to the highly rearranged receptors of adaptive immunity, PRRs are limited in their number, germline-encoded, and evolutionary conserved [35].

The mammalian homolog of Drosophila Toll, TLR 4, was described as the first PRR in mice [36] responding to lipopolysaccharide (LPS) [37] as one of the major components of gram-negative bacteria. Until today, 10 different TLRs have been described in humans and 12 in mouse, detecting distinct PAMPs from bacteria, mycobacteria, viruses, fungi, and parasites, whereas one intact pathogen usually contains different PAMPs activating different PRRs [38]. Downstream signaling of TLRs initiates two major signaling pathways: except for TLR 3, which is TRIF-dependent (TIR domain–containing adaptor-inducing interferon-β–dependent), all TLRs recruit myeloid differentiation protein 88 (MyD88)–dependent signaling.

### TLR4 exerts different effects in different murine models of arterial hypertension

Enforced TLR-signaling (especially TLR4- and MyD88-dependend pathways) has been described in murine hypertension with a high variability in the effects depending on the investigated model. An overview regarding all different TLRs and their impact on arterial hypertension has been described in detail before [39, 40], so we focus on TLR4 and the partially contradictory findings for its role in murine models of arterial hypertension and discuss the sparse human data.

In genetic models of hypertension, spontaneously hypertensive rats showed higher cardiac TLR4 expression [41] whereas anti-TLR4 antibody treatment decreased blood pressure [42]. These studies provide evidence that enhanced TLR4 expression and TLR4 signaling might be linked to the development and maintenance of hypertension. Same trends could be seen in DOCA-salt and aldosterone-salt hypertensive rats [43]. In mice with NG-nitro-l-arginine methyl ester (l-NAME)–induced hypertension, blood pressure was blunted in TLR4 knockout mice compared with wild-type mice [44], supporting the role of TLR4—at least—in this experimental model of arterial hypertension.

For AngII-infused mice, findings are complex and highly dependent of the studied tissue. Dange et al. published evidence that the blockade of brain TLR4 attenuates blood pressure in the AngII model [45]. In contrast to these findings, Singh et al. found that AngII-induced hypertension was not affected in MyD88^−/−^ but in TRIF^mut^ mice [46] and that a full knockout of TLR4 kept a preserved or even enhanced hypertensive response after the infusion of AngII [47]. This in line with findings of Matsuda et al. who could reproduce the results in TL4-deficient mice [48].

**Fig. 1:**
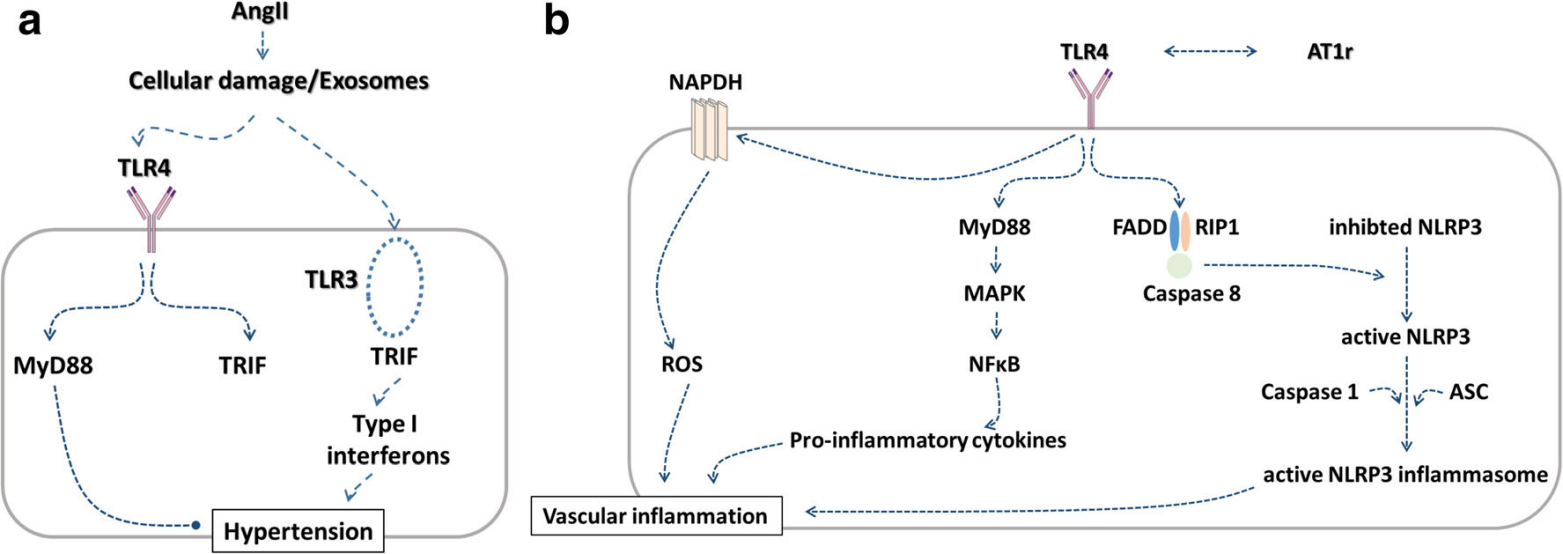
Possible crosstalk between angiotensin II (AngII) and Toll-like receptor 4 (TLR 4) in arterial hypertension. **a** A model proposed by Singh et al. [46, 47] with different effects of TLR 2 and 4 in AngII-induced murine hypertension. The studies propose intrinsic differences between TLR4-TRIF and TLR3-TRIF interactions, where TLR3 directly interacts with TRIF and is required for AngII hypertension, whereas TLR4 activates MyD88, which lowers blood pressure in their studies using AngII-infused mice. **b** AngII acting through AT1 receptors upregulates TLR4. MyD88-dependent intercellular signaling results in MAP-kinase and NFκB activation with consequent increased production of pro-inflammatory mediators, which in turn contributes to hypertension. Additionally, TLR4 can induce NLRP3 inflammasome by activation of a receptor-interacting protein 1 (RIP1)–FAS-associated death domain protein (FADD)–caspase 8. Furthermore, AngII drives the generation of reactive oxygen species through NADPH oxidase. Downstream of all pathways, TLR4 exerts pro-inflammatory effects which can contribute to arterial hypertension. TLR 4, Toll-like receptor 4; AT1r, Angiotensin II receptor type I, NAPDH, nicotinamide adenine dinucleotide phosphate oxidase; MyD88, Myeloid differentiation primary response 88; ROS, reactive oxygen species; MAPK, mitogen-activated protein kinase; FADD, FAS-associated death domain protein; NFκB, nuclear factor “kappa-light-chain-enhancer” of activated B cells; NLRP3, nucleotide-binding oligomerization domain and leucine-rich repeat-containing receptor 3; ASC, apoptosis-associated Speck-like protein containing a caspase-recruitment domain

In TLR3-deficient mice, AngII-induced hypertension was abrogated [47] suggesting the TLR3-TRIF pathway as crucial for a blood pressure increase in the AngII model (Fig. 1a). The divergent findings between a full knockout and a brain-specific blockade might indicate either that the investigated tissue is of importance or that the models are so variable and unstable regarding the phenotype that a comparison is not possible. In cardiomyocytes, a different mechanism with a possible interaction between TLR and AngII signaling was published by Han et al. who supposed a MD2-mediated direct activation of TLR-signaling by AngII via TLR4 [49], whereas other experiments with the AT1R-antagonist Valsartan suggest a crosstalk between AT1R- and TLR4-dependent signaling [50] (Fig. 1b). Regarding the human population, Schneider et al. demonstrated that in a cohort of patients with myocardial infarction, older carriers of a TLR4 single-nucleotide polymorphism have a lower systolic blood pressure and pulse pressure indicating that TLR4 signaling influences age-dependent blood pressure increases [51].

Taken together, there is on the one hand promising murine data regarding TLR4-signaling as a potential pharmaceutical target in essential hypertension. On the other hand, the results are strongly depending on the used murine model and could not have been solidly reproduced in humans yet.

## NLRP3-inflammasome

Another downstream target of the TLR-4 signaling cascade, the cytosolic NOD-, LRR-, and pyrin domain–containing 3-receptor (NLRP3), has attracted attention within the past years, offering a novel possible pharmacological target for different inflammatory diseases [52]. NLRP3 drives the assembly of the inflammasome, leading to caspase 1–dependent activation of interleukin-1β (IL-1 β) cytokines. Crowley et al. demonstrated that renal IL-α and IL-β levels correlate with systolic blood pressure levels [53] and that IL-1R1 deficiency and blockage lowers blood pressure by reducing sodium re-uptake in the kidneys of AngII-infused animals [54].

Besides potassium-dependent pathways of NLRP3 activation, TLR4 can induce NLRP3-inflammasome by activation of a receptor-interacting protein 1 (RIP1)–FAS-associated death domain protein (FADD)–caspase 8 pathway [55]. Krishnan et al. demonstrated that inflammasome activity is crucial in one-side nephrectomized mice treated with DOCA-high-salt diet [56] and interfering with the NLRP3 inflammasome provided antihypertensive effects [57]. The authors conclude that the observed renal inflammation driven by DOCA/high-salt diet is responsible for arterial hypertension in their model and can be prevented by a pharmacological inhibition of the NLRP3 inflammasome as a novel antihypertensive strategy. These publications are in line with other papers, which link high salt intake with macrophage activation and polarization in other tissues [58, 59]. In the bigger picture of risk factors driving vascular dysfunction and atherosclerosis, targeting NLRP3 might offer a second benefit as well. Christ et al. published strong evidence that western diet triggers NLRP3-dependent innate immune reprogramming [60] and therefore exacerbates pro-inflammatory conditions.

## Humoral innate immunity

### Antimicrobial peptides and the complement system

Already beyond the anatomical borders, components of the innate immunity defend the integrity of the body against pathogens. Mucus and plasma are enriched with different classes of secreted antimicrobial peptides. Epithelial and circulating cells release defensins, cationic polypeptides with direct and indirect antimicrobial functions. There are still different hypotheses about the exact mechanisms of their microbe-killing capacities, but all focus on interactions between their molecular structure and the membrane of the microbes [61]. Nassar et al. could describe the presence of α-defensin in human coronary arteries and decrease the contraction of smooth muscle cells in response to the vasoconstrictor phenylephrine via the low-density lipoprotein receptor–related protein/α2-macroglobulin receptor, indicating a direct effect of these peptides on vascular tone and a possible role in hypertension as well [62].

Lysozyme, a glycoside hydrolase catalyzing the hydrolysis of 1,4-beta-linkages between *N*-acetylmuramic acid and *N*-acetyl-d-glucosamine, is the most active component of all antimicrobial peptides. Several studies have already investigated a possible interplay between lysozyme and hypertension. With the intention to find a way to measure markers for subclinical atherosclerosis in an easy approachable and non-invasive way, especially salivary levels of lysozyme and other inflammatory molecules have been investigated within the past years. In two different studies, lysozyme in saliva was associated with hypertension. Qvarnstrom et al. suggested in a study with 500 participants that the top quartile of salivary lysozyme levels is significantly associated with prevalent hypertension [63]; similar findings were described in 259 humans by Labat et al. [64] A complementary trend has already been described for salivary lysozyme and coronary heart disease. The authors of the study interpreted these findings in line with the general paradigm that inflammation is an important part of pathogenesis of hypertension and atherosclerosis without defining the relationship as causal.

The complement system is component of the innate immune system. It is a complex humoral cascade consisting of over 30 soluble and membrane-bound molecules which directly sense and destroy microbial invaders [65] via opsonization and phagocytosis or enhance the cellular immune response via induction of chemokine or cytokine production. The complement cascade is mainly found not only in the circulation but also in tissues [66]. Increased plasma levels of different complement proteins in human patients with hypertension have been described. For instance, Eggström et al. and Bao et al. could find a positive association of elevated blood pressure and prehypertension with blood levels of the complement protein C3 [67, 68]. The same trend has also been seen for C3a [69], C4 [68], and C5a [70] indicating that hypertension induces the activation of complement system and production of complement factors.

In murine models, interfering with the complement system has been shown to provide protective mechanisms especially against hypertensive end-organ damage. Pharmacological inhibition of C5a production significantly reduced cardiac inflammation and remodeling in AngII-induced hypertension [70, 71]. Not only for cardiac end-organ damage but also for hypertension-driven pathologic changes in the kidney, different components of the complement system seem to be crucially involved. Chen et al. published that AngII-infused mice deficient for C5a and C3a receptors did not develop arterial hypertension by a decreased renal macrophage and T cell infiltration [72]; moreover, Zhou et al. showed that C3a activates the RAAS system by induction of epithelial-to-mesenchymal transition in renal epithelial cells [73].

Given the promising evidence of the conducted murine studies, the complement system is a likely target in further advances of hypertension research. Promising data regarding the prevention of hypertensive end-organ damage more than possible blood pressure–lowering effects may be at the center of attention in this field.

## Anatomical barriers

### Visible but often overlooked—the physical barriers gut and skin as the first line of defense in innate immunity

Even though it is the only part of the innate immune system, which is immediately visible for the human eye, skin and mucosal barriers tend to be ignored as innate immune organs. Besides the physical barrier function, the skin and the gut represent the outer interface with the external environment, and are therefore essential organs for the maintenance of physiologic homeostasis and closely related in purpose and function [74]. Within recent years, the skin and the gut have reached growing interest regarding their role in arterial hypertension. More particularly, these additional approaches provide a more physiology-oriented than a molecular biology-oriented approach, which might help to find new treatment strategies for essential hypertension.

### The gastrointestinal barrier—human organ or microbiota zoo?

Under healthy conditions, the gastrointestinal barrier exerts an important role in nourishing the organism by digestion of food, influences the development and function of the mucosal immune system, and is an active endocrine organ. The contribution of the gastrointestinal barrier in arterial hypertension has been linked to the effects of gastrointestinal hormones, which were shown to influence systemic blood pressure levels in different murine models. Especially administration of the incretin hormone glucagon-like peptide-1 significantly attenuated the development of hypertension in Dahl salt-sensitive [75] and spontaneously hypertensive rats [76] and antihypertensive capacities of GLP-1 receptor agonists even could provide promising results in human hypertensive diabetics [77].

Within the last years, the gastrointestinal barrier unexpectedly advanced into the closer focus of attention in arterial hypertension—not by the organ function itself but by the fascinating inhabitants it accommodates. In the introduction of their work about the “Human microbiome project,” Turnbaugh et al. described humans as “supraorganisms composed of human and microbial components,” [78] underlining the undeniable close connection between the human organism in health as well as in disease and the microbes that live inside and on us. As the intestine is the organ of the body which is most densely populated with microorganisms, the major part of this emerging research field is dealing with this site, where the microbiome has a deep impact on human body development and contributes to normal physiology and disease developement [79].

Human microbial colonies in the gastro-intestine are described to be generally stable [80], but can be immediately changed and shaped by a large scale of influences. Not surprisingly, the consumed diet is a major factor for the composition and function of gut microbiota [81, 82]. Recent evidence revealed that certain diseases can be transferred via the microbiome: transferring microbiota from genetically obese mice induced higher amounts of body fat in germ-free mice [83]. These findings indicate that not only the diet has an effect on the microbiome but also the microbiome itself can influence host metabolism, which has been proven in further publications [84].

Several studies have linked gut microbiota and dysbiosis of microbiota with arterial hypertension in mice and men [85, 86]. Karbach et al. showed that germ-free-raised mice without a colonializing microbiome do not develop arterial hypertension and vascular dysfunction in response to AngII [87]. The lifelong absence of gut microbiota protected them from AngII-induced arterial hypertension and vascular dysfunction by reduced vascular inflammation and diminished production of reactive oxygen species.

Studies in different murine models of arterial hypertension could show that hypertensive animals have alterations in their microbiome and that it is possible to transfer hypertension by transferring microbiota from hypertensive to formerly normotensive animals [85, 88]. Even more interesting, dietary interventions, which affected the microbial colonization of the gut, modified blood pressure, and protected the animals from hypertension-related end-organ damage in murine models of arterial hypertension [89, 90]. Vice-versa, high-salt diet–driven changes of the gut microbiome induced Th17 cells and caused hypertension in mice, which could successfully be restored by probiotic treatment [91].

Taken together, the studies indicate that the gut microbiome is a fascinating field of research not only to develop a deeper understanding in microbiota host or interactions but also regarding a novel and profitable drug target in arterial hypertension.

### Skin, sodium, and sodium-dependent hypertension

Besides its role as an outer barrier, the skin, especially skin glycose-aminoglycans, was detected as large compartment for water-free sodium storage regulating body sodium balance independently of renal function [92, 93]. These findings started to challenge the concept of pressure-natriuresis, an almost 50-year-old theory explaining the causal connection between high-salt intake and high blood pressure.

The pressure-natriuresis approach by Guyton et al. [94] described a first concept of how salt intake regulates blood pressure which led the field for more than half a century. It supposes that direct changes in salt and water intake and the following excretion drive the direction and change in arterial pressure to maintain body salt-water balance. By introducing the skin as the crucial compartment for long-term sodium storage and skin tissue sodium content as a detectable signature which is also non-invasively detectable in human hypertensive patients [95–97], Titze et al. indicated that the single-compartment concept of pressure-natriuresis for sodium excretion and blood pressure control might fall short. In a human long-term balance study, they could prove more evidence by detecting continuous fluctuations in sodium excretion, independent of sodium intake, indicating that sodium is stored and released in the skin without a direct impact on parallel water storage or blood pressure increase [98]. Helle et al. contributed further functional aspects of skin sodium storage for the genesis of arterial hypertension by showing that skin arterioles from rats fed a high-salt diet had increased contractility in response to AngII [99].

**Fig. 2:**
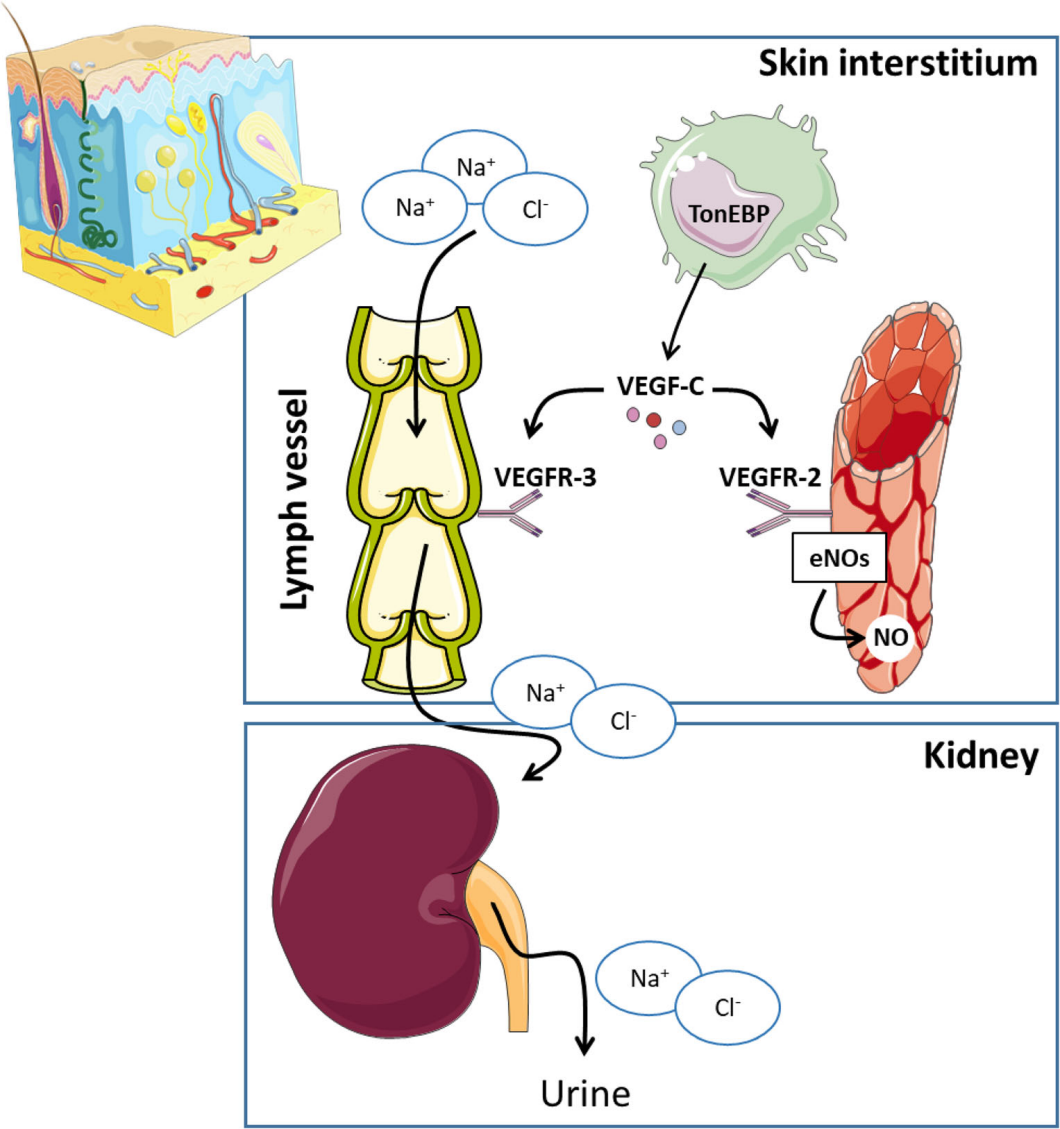
Osmotically inactive Na storage in the skin contributes to body sodium and blood pressure regulation. Interstitial electrolyte balance relies on extrarenal regulatory mechanisms within the skin interstitium. Macrophages act as interstitial osmosensors that regulate local electrolyte composition via a tonicity enhancer binding protein/vascular endothelial growth factor-C (TonEBP/VEGF-C)–dependent mechanism [100]. The subsequent modulation of the lymph capillary network in the skin drives clearance of interstitial electrolytes from the interstitium into the bloodstream for renal clearance. Failure of this physiological extrarenal regulatory mechanism leads to a salt-sensitive blood pressure response. eNOS, endothelial nitric oxide synthase; VEGFR, vascular endothelial growth factor receptor; TonEBP, tonicity enhancer binding protein

The detected storage of sodium in the skin thereby does not represent a random or incidental process at all. Beyond keratinocytes and extracellular matrix components, the skin barrier accommodates a variety of innate immune cells exerting phagocytic as well as regulatory functions: dendritic cells (DCs), natural killer (NK) cells, innate lymphoid cells (ILCs), mast cells, γδ-T cells, and myelomonocytic cells. These cellular representatives of the innate immune system, in particular tissue-resident macrophages, play a key role in sensing the interstitial, extracellular electrolyte levels in the skin (Fig. 2). Via the expression of tonicity-responsive enhancer binding protein (TONEBP), skin macrophages may modify electrolyte clearance through an extension of the skin lymphatic capillaries, which is part of the body blood pressure regulation [100].

Taken together, the skin is a key component regarding the innate immune system and mechanisms of hypertension. At cutaneous tissue level, macrophages modulate the interstitial salt storage and release by changing the lymph capillary network in the skin, which results in a kidney-like lymphatic counter-current system. As the body’s largest reservoir for sodium storage, it is—next to the kidney—a crucial organ for regulating the body salt-water balance and arterial blood pressure.

## Conclusion

Innate immune cells are important drivers and modulators in the pathophysiology of hypertension on many levels. It has never been tested specifically, whether a pharmacologic intervention that would impact on anatomical barriers or other components’ innate immune system is effective to treat human arterial hypertension or prevent emergence or exacerbation of high blood pressure. An exception is concomitant autoimmune disease such as psoriasis or rheumatoid arthritis; patients afflicted by these diseases who were treated with mycophenolate mofetil, an inhibitor of inosine monophosphate dehydrogenase and suppressant of T and B cell proliferation [101], or the monoclonal TNF-α-antibody, infliximab [102], had reductions in blood pressure. There seems to be a strong link between genes favoring an enhanced status of inflammasome activation, IL-1β levels, arterial hypertension, and reduced longevity [103]. Whether anti-IL-1β helps to reduce increased blood pressure, however, is unclear. Secondary analyses from the CANTOS trial [104, 105] at least suggest that the IL-1β antibody canakinumab, which was effective to prevent cardiovascular events in patients with established coronary artery disease, was neutral with regard to arterial hypertension [106], although this trial also revealed that hsCRP level increases with increasing tertiles of blood pressure values at baseline. Whether arterial hypertension can specifically be treated by an anti-inflammatory therapy [107] remains to be tested in randomized controlled trials.

## Data Availability

Not applicable.
